# Transferring arbitrary *d*-dimensional quantum states of a superconducting transmon qudit in circuit QED

**DOI:** 10.1038/s41598-017-07225-5

**Published:** 2017-08-01

**Authors:** Tong Liu, Qi-Ping Su, Jin-Hu Yang, Yu Zhang, Shao-Jie Xiong, Jin-Ming Liu, Chui-Ping Yang

**Affiliations:** 1 Department of Physics, Hangzhou Normal University, Hangzhou, Zhejiang 310036 China; 2 State Key Laboratory of Precision Spectroscopy, Department of Physics, East China Normal University, Shanghai, 200062 China

## Abstract

A qudit (*d*-level quantum system) has a large Hilbert space and thus can be used to achieve many quantum information and communication tasks. Here, we propose a method to transfer arbitrary *d*-dimensional quantum states (known or unknown) between two superconducting transmon qudits coupled to a single cavity. The state transfer can be performed by employing resonant interactions only. In addition, quantum states can be deterministically transferred without measurement. Numerical simulations show that high-fidelity transfer of quantum states between two superconducting transmon qudits (*d* ≤ 5) is feasible with current circuit QED technology. This proposal is quite general and can be applied to accomplish the same task with natural or artificial atoms of a ladder-type level structure coupled to a cavity or resonator.

## Introduction

Many quantum information and communication tasks are usually based on qubits (two-level quantum systems), but the use of qudits (*d*-level quantum systems) can optimize some quantum computations^1, 2^, enhance the security of quantum cryptography^3, 4^, realize bipartite entanglement^5^, verify entropic inequalities^6^, implement quantum algorithms^7^, and simplify the construction of quantum logic gates^8, 9^. In addition, manipulation and measurement of a superconducting phase qudit state or preparation and control of a transmon qudit has been reported in experiments^10, 11^. Moreover, population transfer of a three-level transmon qudit for *d* = 3, via stimulated Raman adiabatic passage, has been experimentally demonstrated recently^12^.

During the past years, superconducting qubits/qudits have been paid intensive attention in quantum information and quantum computation due to their significantly increased coherence times, controllability and scalability^13–21^. Superconducting qubits/qudits based on Josephson junctions are mesoscopic element circuits that behave like “artificial atoms”, whose level spacings can be rapidly (within 1–3 ns) adjusted by varying external control parameters (e.g., magnetic flux applied to the superconducting loop of a superconducting phase, transmon, Xmon, or flux qubit/qudit; see, e.g., refs 18, 22–24).

Circuit quantum electrodynamics (circuit QED) is analogue of cavity QED, which has been considered as one of the most promising candidates for quantum information processing (QIP)^14, 15, 25–27^. The strong-coupling or ultrastrong-coupling regime with a superconducitng qubit coupled to a microwave resonator has been experimentally realized in circuit QED^28–31^. Using superconducting qubits coupled to a single cavity or resonator, many theoretical proposals have been presented for realizing quantum gates and entanglement^25–27, 32–37^. Quantum effects and operations have been experimentally demonstrated with superconducting qubits in circuit QED, including demonstration of two-and three-qubit quantum gates^38–42^, realization of two-and three-qubit entanglement^23, 43^, observation of Raman coherence effects^44^, and suppression of dephasing by qubit motion^45^. Moreover, a number of theoretical proposals have been proposed for realizing quantum state transfer (QST) between two superconducting qubits through a cavity^26, 33, 46–51^. The QST between two superconducting qubits has been experimentally demonstrated in circuit QED^52–55^.

The qudit-to-qudit QST plays a vital role in high-dimensional quantum communication and QIP. Transfer of high-dimensional photon states through a cavity array was previously proposed in refs 56 and 57. In addition, probabilistic transfer of high-dimensional quantum states between particles via a spin chain has been studied^58, 59^. Moreover, a method has been proposed for transferring quantum states between two superconducting transmon qutrits via an adjustable inductive coupling^60^, and an approach has been presented for transferring quantum states between two superconducting flux qutrits coupled to two resonators or cavities^61^. Here, qutrit refer to a three-level quantum system or a qudit for *d* = 3. Refs 60 and 61 only work for QST between two *qutrits* and ref. 61 requires the use of *two* resonators or cavities coupled to each qutrit. Note that in a circuit consisting of two or more resonators, the inter-resonator crosstalk is inevitable^62^, which degrades the performance of quantum operations and the fidelity of various quantum states.

Different from the previous works, we here propose a method to transfer arbitrary *d*-dimensional quantum states (known or unknown) between two superconducting transmon qudits coupled to a single cavity. As shown below, this proposal has the following advantages: (i) The experimental setup is very simple because only one cavity is used; (ii) Since employing a single cavity, the inter-cavity crosstalk is avoided; (iii) The QST can be realized by using qudit-cavity and qudit-pulse resonant interactions only; (iv) The QST can be deterministically achieved without measurement; and (v) The method can in principle be applied to transfer arbitrary *d*-dimensional quantum states between two *d*-level qudits for any positive integer *d*. This proposal is quite general and can be applied to accomplish the same task with “ladder-type level structure” natural or artificial atoms coupled to a cavity or resonator.

In this work, we will show how to transfer arbitrary quantum states between two superconducting transmon qudits coupled to a cavity or resonator. We will also discuss the experimental feasibility of this proposal, by considering a setup of two transmon qudits coupled to a 3D cavity and numerically calculating the fidelity for the QST between two transom qudits for *d* ≤ 5.

## Results

### Quantum state transfer between two superconducting transmon qudits

**Figure 1:**
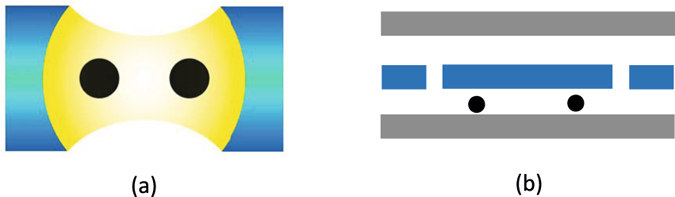
(**a**) Setup for two superconducting transmon qudits embedded in a 3D cavity. (**b**) Setup for two superconducting transmon qudits coupled to a 1D transmission line resonator. A dark dot represents a superconducting transmon qudit.

Our system, shown in Fig. 1, consists of two superconducting transmon qudits 1 and 2 embedded in a 3D microwave cavity or coupled to a 1D resonator. In reality, the *d* involved in QIP may not be a large number. Thus, as an example, we will explicitly show how to transfer quantum states between two transmon qudits for *d* ≤ 5. We then give a brief discussion on how to extend the method to transfer arbitrary *d*-dimensional quantum states between two *d*-level transmon qudits for any positive integer *d*.

**Figure 2:**
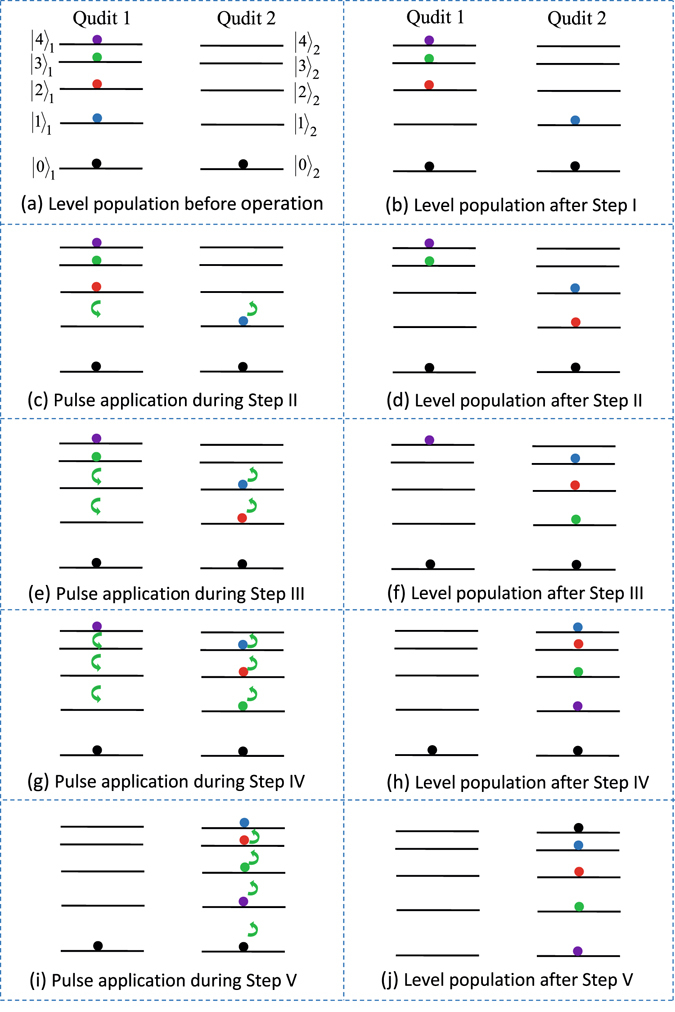
The color circles indicate the occupied energy levels. Each green arrow represents a classical pulse, which is resonant with the transition between the two neighbor levels close to each green arrow. In (**e**) and (**g**), the sequence for applying the pulses is from top to bottom, and the lower pulses are turned on after the upper pulses are switched off. In (**i**), the sequence for applying the pulses is from bottom to top, and the upper pulses are turned on after the lower pulses are switched off. For the details on the applied pulses, see the descriptions given in the text. Note that in (**a**–**j**), the left levels are for qudit 1 while the right levels are for qudit 2. For simplicity, we here consider the case that the spacings between adjacent levels become narrow as the levels move up, which is actually unnecessary.

A transmon qudit has a ladder-type level structure^63^. We here label the *d* levels as $|0\rangle ,|1\rangle ,|2\rangle ,\mathrm{...}\,{\rm{and}}\,|d-1\rangle$ (Fig. 2 for *d* = 5). For a ladder-type level structure, the transition between adjacent levels is allowed but the transition between non-adjacent levels is forbidden or very weak. In the following, the transition frequency between two adjacent levels $|l-1\rangle \,{\rm{and}}\,|l\rangle$ of each qudit is labeled as *ω*_(*l* − 1)*l*_ (*l* = 1, 2, …, *d* − 1). The initial phase, duration, and frequency of the pulses are denoted as {*ϕ*, *t*, *ω*}. For simplicity, we set the same Rabi frequency Ω for each pulse, which can be readily achieved by adjusting the pulse intensity. Here and below, qudit (qudits) refers to transmon qudit (qudits).

### Case for *d* = 5

The five levels of qudits are labeled as $|0\rangle ,|1\rangle ,|2\rangle ,|3\rangle ,\,{\rm{and}}\,|4\rangle$ (Fig. 2). Assume that qudit 1 is initially in an arbitrary quantum state ${\sum }_{l=0}^{4}{c}_{l}{|l\rangle }_{1}$ (known or unknown) with level populations illustrated in Fig. 2(a), qudit 2 is initially in the ground state |0〉_2_, and the cavity is initially in the vacuum state |0〉_*c*_. Here and below, *c*_*l*_ is a normalized coefficient.

To begin with, the level spacings of the qudits need to be adjusted to have the cavity resonant with the |0〉 ↔ |1〉 transition of each qudit. The procedure for implementing the QST from qudit 1 to qudit 2 is described as follows:

Step I. Let the cavity resonant with the |0〉 ↔ |1〉 transition of each qudit described by Hamiltonian (14) (see Section Methods below). According to Eq. (15), after an interaction time *t*_1_ = *π*/$(\sqrt{2}g)$, one has the state transformation

1 $${|1\rangle }_{1}{|0\rangle }_{c}{|0\rangle }_{2}\to -{|0\rangle }_{1}{|0\rangle }_{c}{|1\rangle }_{2},$$

which shows that the cavity remains in the vacuum state after the qudit-cavity interaction. Thus, the initial state ${\sum }_{l=0}^{4}{c}_{l}{|l\rangle }_{1}\otimes {|0\rangle }_{2}$ of the two qudits becomes

2 $$({c}_{0}{|0\rangle }_{1}+{c}_{2}{|2\rangle }_{1}+{c}_{3}{|3\rangle }_{1}+{c}_{4}{|4\rangle }_{1})\mathrm{|0}{\rangle }_{2}-{c}_{1}{|0\rangle }_{1}{|1\rangle }_{2}\mathrm{.}$$

Equation (2) shows that the population of the level |1〉 of qudit 1 is transferred onto the level |1〉 of qudit 2 [Fig. 2(b)].

Step II. Apply a pulse of {*π*/2,*π*/2Ω,*ω*_12_} to qudit 1 while a pulse of {−*π*/2, *π*/2Ω, *ω*_12_} to qudit 2 [Fig. 2(c)]. According to Eq. (17), the pulses lead to ${|2\rangle }_{1}\to {|1\rangle }_{1}$ and ${|1\rangle }_{2}\to {|2\rangle }_{2}$. Thus, the state (2) becomes

3 $$({c}_{0}{|0\rangle }_{1}+{c}_{2}{|1\rangle }_{1}+{c}_{3}{|3\rangle }_{1}+{c}_{4}{|4\rangle }_{1})\mathrm{|0}{\rangle }_{2}-{c}_{1}{|0\rangle }_{1}{|2\rangle }_{2}\mathrm{.}$$

For ${\rm{\Omega }}\gg g$, the interaction between the cavity and the qudits can be neglected during the pulse. Now let the cavity resonant with the |0〉 ↔ |1〉 transition of each qudit for an interaction time *t*_2_ = *π*/$(\sqrt{2}g)$, to obtain the state transformation (1). Hence, the state (3) becomes

4 $$({c}_{0}{|0\rangle }_{1}+{c}_{3}{|3\rangle }_{1}+{c}_{4}{|4\rangle }_{1}){|0\rangle }_{2}+(-\,{c}_{2}{|1\rangle }_{2}-{c}_{1}{|2\rangle }_{2}){|0\rangle }_{1},$$

which shows that the populations for the levels |1〉 and |2〉 of qudit 1 are transferred onto the levels |2〉 and |1〉 of qudit 2, respectively [Fig. 2(d)].

Step III. Apply a pulse of {*π*/2, *π*/2Ω, *ω*_23_} and then a pulse of {*π*/2, *π*/2Ω, *ω*_12_} to qudit 1, while a pulse of {−*π*/2, *π*/2Ω, *ω*_23_} and then a pulse of {−*π*/2, *π*/2Ω, *ω*_12_} to qudit 2 [Fig. 2(e)]. The pulses result in the transformations |3〉_1_ → |1〉_1_ (via |3〉_1_ → |2〉_1_ → |1〉_1_), |2〉_2_ → |3〉_2_ and |1〉_2_ → |2〉_2_. Thus, the state (4) becomes

5 $$({c}_{0}{|0\rangle }_{1}+{c}_{3}{|1\rangle }_{1}+{c}_{4}{|4\rangle }_{1})\mathrm{|0}{\rangle }_{2}+(-{c}_{2}{|2\rangle }_{2}-{c}_{1}{|3\rangle }_{2}){|0\rangle }_{1}\mathrm{.}$$

Let the cavity resonant with the |0〉 ↔ |1〉 transition of each qudit for an interaction time *t*_3_ = *π*/$(\sqrt{2}g)$, to achieve the state transformation (1). Thus, the state (5) becomes

6 $$({c}_{0}{|0\rangle }_{1}+{c}_{4}{|4\rangle }_{1})\mathrm{|0}{\rangle }_{2}+(-{c}_{3}{|1\rangle }_{2}-{c}_{2}{|2\rangle }_{2}-{c}_{1}{|3\rangle }_{2}){|0\rangle }_{1},$$

which shows that the populations for the levels |1〉, |2〉, and |3〉 of qudit 1 are transferred onto the levels |3〉, |2〉, and |1〉 of qudit 2, respectively [Fig. 2(f)].

Step IV. Apply pulses of {*π*/2, *π*/2Ω, *ω*_34_}, {*π*/2, *π*/2Ω, *ω*_23_} and then {*π*/2, *π*/2Ω, *ω*_12_} to qudit 1 while pulses of {−*π*/2, *π*/2Ω, *ω*_34_}, {−*π*/2, *π*/2Ω, *ω*_23_} and then {−*π*/2, *π*/2Ω, *ω*_12_} to qudit 2 [Fig. 2(g)], which leads to the transformations |4〉_1_ → |1〉_1_ (via|4〉_1_ → |3〉_1_ → |2〉_1_ → |1〉_1_), |3〉_2_ → |4〉_2_, |2〉_2_ → |3〉_2_ and |1〉_2_ → |2〉_2_. Hence, the state (6) becomes

7 $$({c}_{0}{|0\rangle }_{1}+{c}_{4}{|1\rangle }_{1})\mathrm{|0}{\rangle }_{2}+(-{c}_{3}{|2\rangle }_{2}-{c}_{2}{|3\rangle }_{2}-{c}_{1}{|4\rangle }_{2}){|0\rangle }_{1}\mathrm{.}$$

Let the cavity resonant with the |0〉 ↔ |1〉 transition of each qudit for an interaction time *t*_4_ = *π*/$(\sqrt{2}g)$, to have the state transformation (1). Thus, the state (7) changes

8 $$({c}_{0}{|0\rangle }_{2}-{c}_{4}{|1\rangle }_{2}-{c}_{3}{|2\rangle }_{2}-{c}_{2}{|3\rangle }_{2}-{c}_{1}{|4\rangle }_{2}){|0\rangle }_{1},$$

which shows that the populations for the levels ${|1\rangle }_{1},{|2\rangle }_{1},{|3\rangle }_{1},$ and |4〉_1_ of qudit 1 have been transferred onto the levels ${|4\rangle }_{2},{|3\rangle }_{2},{|2\rangle }_{2},$ and |1〉_2_ of qudit 2, respectively [Fig. 2(h)]. After this step of operation, to maintain the state (8), the level spacings of the qudits need to be adjusted so that the qudits are decoupled from the cavity.

Step V. By sequentially applying pulses of {−*π*/2, *π*/2Ω, *ω*_01_}, {−*π*/2, *π*/2Ω, *ω*_12_}, {−*π*/2, *π*/2Ω, *ω*_23_}, and then {−*π*/2, *π*/2Ω, *ω*_34_} to qudit 2 [Fig. 2(i)], we obtain the state transformations ${|0\rangle }_{2}\to {|4\rangle }_{2}$ (via ${|0\rangle }_{2}\to {|1\rangle }_{2}\to {|2\rangle }_{2}\to {|3\rangle }_{2}\to {|4\rangle }_{2}$), ${|1\rangle }_{2}\to -{|0\rangle }_{2},$ ${|2\rangle }_{2}\to -{|1\rangle }_{2},$ ${|3\rangle }_{2}\to -{|2\rangle }_{2},$ and ${|4\rangle }_{2}\to -{|3\rangle }_{2}\mathrm{.}$ Hence, the state (8) becomes

9 $$({c}_{4}{|0\rangle }_{2}+{c}_{3}{|1\rangle }_{2}+{c}_{2}{|2\rangle }_{2}+{c}_{1}{|3\rangle }_{2}+{c}_{0}{|4\rangle }_{2}){|0\rangle }_{1}\mathrm{.}$$

The result (9) shows that an arbitrary quantum state ${\sum }_{l=0}^{4}\,{c}_{l}|l{\rangle }_{1}$ of qudit 1 has been transferred onto qudit 2 via the population transfer from the five levels $\{{|0\rangle }_{1},{|1\rangle }_{1},{|2\rangle }_{1},{|3\rangle }_{1},{|4\rangle }_{1}\}$ of qudit 1 to the five levels $\{{|4\rangle }_{2},{|3\rangle }_{2},{|2\rangle }_{2},{|1\rangle }_{2},{|0\rangle }_{2}\}$ of qudit 2, respectively [Fig. 2(j)].

### Case for *d* = 4 and *d* = 3

From the above description, it can be found that by performing the operations of steps I, II, and III above, and then by sequentially applying pulses of {−*π*/2, *π*/2Ω, *ω*_01_}, {−*π*/2, *π*/2Ω, *ω*_12_}, and {−*π*/2, *π*/2Ω, *ω*_23_} to qudit 2, we can obtain the state transformation ${\sum }_{l=0}^{3}\,{c}_{l}|l{\rangle }_{1}\otimes \mathrm{|0}{\rangle }_{2}\to \mathrm{|0}{\rangle }_{1}\otimes ({c}_{0}{|3\rangle }_{2}+{c}_{1}{|2\rangle }_{2}+{c}_{2}{|1\rangle }_{2}+{c}_{3}{|0\rangle }_{2}),$ which implies that the QST for *d* = 4 is implemented, i.e., an arbitrary quantum state of qudit 1 is transferred onto qudit 2 via the population transfer from the four levels $\{{|0\rangle }_{1},{|1\rangle }_{1},{|2\rangle }_{1},{|3\rangle }_{1}\}$ of qudit 1 to the four levels $\{{|3\rangle }_{2},{|2\rangle }_{2},{|1\rangle }_{2},{|0\rangle }_{2}\}$ of qudit 2, respectively.

By performing the operations of steps I and II above, followed by applying pulses of {−*π*/2, *π*/2Ω, *ω*_01_} and then {−*π*/2, *π*/2Ω, *ω*_12_} to qudit 2, the state transformation ${\sum }_{l=0}^{2}\,{c}_{l}|l{\rangle }_{1}\otimes \mathrm{|0}{\rangle }_{2}\to \mathrm{|0}{\rangle }_{1}\otimes ({c}_{0}{|2\rangle }_{2}+{c}_{1}{|1\rangle }_{2}+{c}_{2}{|0\rangle }_{2})$ can be achieved, which shows that the QST for *d* = 3 (i.e., the QST between two qutrits) is realized, i.e., an arbitrary quantum state of qudit 1 is transferred onto qudit 2 via transferring the populations of the three levels $\{{|0\rangle }_{1},{|1\rangle }_{1},{|2\rangle }_{1}\}$ of qudit 1 to the three levels $\{{|2\rangle }_{2},{|1\rangle }_{2},{|0\rangle }_{2}\}$ of qudit 2, respectively.

### Case for any positive integer *d*

By examining the operations introduced in subsection A (i.e., QST for *d* = 5), one can easily find that an arbitrary *d*-dimensional quantum state can be transferred between two *d*-level qudits for any positive integer *d*, through the following *d* operational steps. The first operational step is the same as that described in step 1 above. For the *l* ^*th*^ operational step (1 < *l* < *d*), *l* − 1 pulses of {*π*/2, *π*/2Ω, *ω*_(*l* − 1)*l*_}, $\cdots$, {*π*/2, *π*/2Ω, *ω*_23_}, and {*π*/2, *π*/2Ω, *ω*_12_} should be applied to qudit 1 in turn (from left to right), while other *l* − 1 pulses of {−*π*/2, *π*/2Ω, *ω*_(*l* − 1)*l*_}, $\cdots$, {−*π*/2, *π*/2Ω, *ω*_23_}, and {−*π*/2, *π*/2Ω, *ω*_12_} should be applied to qudit 2 in sequence (from left to right), followed by each qudit simultaneously resonantly interacting with the cavity for an interaction time *t* = *π*/$(\sqrt{2}g)$. One can easily check that after the first *d* − 1 steps of operation, the following state transformation can be obtained ${\sum }_{l=0}^{d-1}\,{c}_{l}|l{\rangle }_{1}\otimes \mathrm{|0}{\rangle }_{2}\to \mathrm{|0}{\rangle }_{1}\otimes ({c}_{0}{|0\rangle }_{2}-{\sum }_{l=1}^{d-1}\,{c}_{l}|d-l{\rangle }_{2}),$ which can further turn into

10 $$\sum _{l=0}^{d-1}\,{c}_{l}|l{\rangle }_{1}\otimes \mathrm{|0}{\rangle }_{2}\to \mathrm{|0}{\rangle }_{1}\otimes ({c}_{0}{|d-1\rangle }_{2}+{c}_{1}{|d-2\rangle }_{2}+\cdots +{c}_{d-1}{|0\rangle }_{2}),$$

by sequentially applying pulses of {−*π*/2, *π*/2Ω, *ω*_01_}, {−*π*/2, *π*/2Ω, *ω*_12_}, .., and then {−*π*/2, *π*/2Ω, *ω*_(*d* − 2)(*d* − 1)_} to qudit 2 (i.e., the last step of operation). The result (10) implies that an arbitrary *d*-dimensional quantum state of qudit 1 (known or unknown) has been transferred onto qudit 2 through the population transfer from the *d* levels $\{{|0\rangle }_{1},{|1\rangle }_{1},{|2\rangle }_{1},\ldots ,{|d-2\rangle }_{1},{|d-1\rangle }_{1}\}$ of qudit 1 to the *d* levels $\{{|d-1\rangle }_{2},{|d-2\rangle }_{2},\ldots ,{|2\rangle }_{2},{|1\rangle }_{2},{|0\rangle }_{2}\}$ of qudit 2, respectively.

#### Possible experimental implementation

For an experimental implementation, let us now consider a setup of two superconducting transmon qudits embedded in a 3D cavity. This architecture is feasible in the state-of-the-art superconducting setup as demonstrated recently in ref. 11. For simplicity, we consider QST between the two transmon qudits 1 and 2 for *d* ≤ 5. As an example, suppose that the state of qudit 1 to be transferred is: (i) $\frac{1}{\sqrt{3}}{\sum }_{l=0}^{2}\,|l{\rangle }_{1}$ for *d* = 3, (ii) $\frac{1}{2}{\sum }_{l=0}^{3}\,|l{\rangle }_{1}$ for *d* = 4, and (iii) $\frac{1}{\sqrt{5}}{\sum }_{l=0}^{4}\,|l{\rangle }_{1}$ for *d* = 5.

We take into account the influence of the unwanted coupling of the cavity with the $|1\rangle \leftrightarrow |2\rangle$ transition. The Hamiltonian *H*_*I*, 1_ is thus modified as

11 $${H}_{I\mathrm{,1}}^{^{\prime} }={H}_{I\mathrm{,1}}+{\varepsilon }_{1},$$

where *ε*_1_ describes the unwanted off-resonant coupling between the cavity and the $|1\rangle \leftrightarrow |2\rangle$ transition of each qudit, which is given by ${\varepsilon }_{1}={\sum }_{j=1}^{2}\,{\tilde{g}}_{j}[a{\sigma }_{{\mathrm{12,}}_{j}}^{+}{e}^{i({\omega }_{12}-{\omega }_{c})t}+h\mathrm{.}c\mathrm{.]}$ where ${\tilde{g}}_{j}$ is the coupling constant between the cavity and the |1〉 ↔ |2〉 transition of qudit *j* (*j* = 1, 2), *ω*_*c*_ is the frequency of the cavity and ${\sigma }_{{\mathrm{12,}}_{j}}^{+}\mathrm{=|2}{\rangle }_{j}\langle \mathrm{1|.}$ For a transmon qudit, one has ${\tilde{g}}_{j}\sim \sqrt{2}{g}_{j}$ ^63^.

We also consider the effect of the unwanted couplings of the pulse with the two adjacent $|l-2\rangle \leftrightarrow |l-1\rangle$ and $|l\rangle \leftrightarrow |l+1\rangle$ transitions, when the pulse is resonant with the $|l-1\rangle \leftrightarrow |l\rangle$ transition of each qudit. Here and below, *l* ∈ {1, 2, 3, 4} for *d* = 5, *l* ∈ {1, 2, 3} for *d* = 4, and *l* ∈ {1, 2} for *d* = 3. After this consideration, the Hamiltonian *H*_*I*, *l*_ is modified as

12 $${H}_{I,l}^{^{\prime} }={H}_{I,l}+{H}_{I\mathrm{,1}}^{^{\prime} }+{\varepsilon }_{l},$$

where *ε*_*l*_ describes the unwanted off-resonant couplings of the pulse with the $|l-2\rangle \leftrightarrow |l-1\rangle$ and $|l\rangle \leftrightarrow |l+1\rangle$ transitions of each qudit, during the pulse resonant with the $|l-1\rangle \leftrightarrow |l\rangle$ transition of each qudit [i.e., the pulse frequency is equal to *ω*_(*l* − 1)*l*_]. Here, *ε*_*l*_ is given by

$({\rm{\Omega }}/\sqrt{2})\{{e}^{-i\phi }{e}^{-i[{\omega }_{(l-\mathrm{1)}l}-{\omega }_{(l-\mathrm{2)(}l-\mathrm{1)}}]t}|l-2\rangle \langle l-1|+h\mathrm{.}c\mathrm{.}\}$ $+\sqrt{2}{\rm{\Omega }}\{{e}^{i\phi }{e}^{i[{\omega }_{(l-\mathrm{1)}l}-{\omega }_{l(l+\mathrm{1)}}]t}|l\rangle \langle l+1|+h\mathrm{.}c\mathrm{.}\}$ ^63^. Note that the effect of the cavity-qudit interaction during the pulse application is also considered here, which is described by the ${H}_{I\mathrm{,1}}^{^{\prime} }\mathrm{.}$

For a transmon qudit, the transition between non-adjacent levels is forbidden or very weak^63^. Thus, the couplings of the cavity/pulses with the transitions between non-adjacent levels can be neglected. In addition, the spacings between adjacent levels for a transmon qudit become narrow as the levels move up (Fig. 2). Therefore, the detunings between the cavity frequency and the transition frequencies for adjacent levels (e.g., levels |1〉 and |2〉, levels |2〉 and |3〉, levels |3〉 and |4〉, etc.) increase when the levels go up. As a result, when compared with the coupling effect of the cavity with the $|1\rangle \leftrightarrow |2\rangle$ transition, the coupling effect of the cavity with the transitions for other adjacent levels is negligibly small, which is thus not considered in the numerical simulation for simplicity. For similar reasons, when the pulse is resonant with the $|l-1\rangle \leftrightarrow |l\rangle$ transition of each qudit, the coupling effect of the pulses with the transitions between other adjacent levels is weak and thus we only consider the effect of the coupling of the pulse with the two adjacent $|l-2\rangle \leftrightarrow |l-1\rangle$ and $|l\rangle \leftrightarrow |l+1\rangle$ transitions.

When the dissipation and dephasing are included, the dynamics of the lossy system is determined by the following master equation

13 $$\begin{array}{rcl}\frac{d\rho }{dt} & = & -\,i[H^{\prime} ,\rho ]+\kappa  {\mathcal L} [a]\\  &  & +\sum _{j=1}^{2}\sum _{l=1}^{d-1}\{{\gamma }_{(l-1)l{,}_{j}} {\mathcal L} [{\sigma }_{(l-1)l{,}_{j}}^{-}]\}\\  &  & +\sum _{j=1}^{2}\sum _{l=1}^{d-1}\{{\gamma }_{\phi l{,}_{j}}({\sigma }_{ll{,}_{j}}\rho {\sigma }_{ll{,}_{j}}-{\sigma }_{ll{,}_{j}}\rho \mathrm{/2}-\rho {\sigma }_{ll{,}_{j}}\mathrm{/2})\},\end{array}$$

where *d* ∈ {3, 4, 5}, *H*′ is the modified Hamiltonian ${H}_{I\mathrm{,1}}^{^{\prime} }$ or ${H}_{I,l}^{^{\prime} }$ given above, ${\sigma }_{(l-1)l{,}_{j}}^{-}={|l-1\rangle }_{j}\langle l|,{\sigma }_{ll{,}_{j}}={|l\rangle }_{j}\langle l|,$ and ${\mathcal L} [{\rm{\Lambda }}]={\rm{\Lambda }}\rho {{\rm{\Lambda }}}^{+}-{{\rm{\Lambda }}}^{+}{\rm{\Lambda }}\rho \mathrm{/2}-\rho {{\rm{\Lambda }}}^{+}{\rm{\Lambda }}\mathrm{/2}$ with ${\rm{\Lambda }}=a,{\sigma }_{(l-1)l{,}_{j}}^{-}\mathrm{.}$ Here, *κ* is the photon decay rate of the cavity. In addition, ${\gamma }_{(l-1)l{,}_{j}}$ is the energy relaxation rate of the level |*l*〉 for the decay path $|l\rangle \to |l-1\rangle$ and ${\gamma }_{\phi l{,}_{j}}$ is the dephasing rate of the level |*l*〉 of qudit *j* (*j* = 1, 2).

The fidelity of the operation is given by ${\mathcal F} =\sqrt{\langle {{\psi }}_{id}|\rho |{{\psi }}_{id}\rangle }$, where $|{\psi }_{id}\rangle$ is the output state of an ideal system (i.e., without dissipation and dephasing considered), which is given by: (i) $|{\psi }_{id}\rangle =|0{\rangle }_{1}\mathrm{|0}{\rangle }_{c}\otimes \mathrm{(1/}\sqrt{3}){\sum }_{l=0}^{2}\,|l{\rangle }_{2}$ for *d* = 3, (ii) $|{\psi }_{id}\rangle =\mathrm{|0}{\rangle }_{1}\mathrm{|0}{\rangle }_{c}\otimes \mathrm{(1/2)}{\sum }_{l=0}^{3}|l{\rangle }_{2}$ for *d* = 4, and (iii) $|{\psi }_{id}\rangle =\mathrm{|0}{\rangle }_{1}\mathrm{|0}{\rangle }_{c}\otimes \mathrm{(1/}\sqrt{5}){\sum }_{l=0}^{4}\,|l{\rangle }_{2}$ for *d* = 5. Note that *ρ* is the final density operator of the system when the operation is performed in a realistic situation.

Without loss of generality, consider identical transmon qudits. In this case, the decoherence rates are the same for each qudit and thus the subscript *j* involved in the decoherence rates above can be omitted. According to ref. 11, we choose *ω*_01_/2*π* = 4.97 GHz, (*ω*_01_ − *ω*_12_)/2*π* = 275 MHz, (*ω*_12_ − *ω*_23_)/2*π* = 309 MHz, and (*ω*_23_ − *ω*_34_)/2*π* = 358 MHz. The decoherence times for the qudits and the cavity, used in the numerical calculation, are as follows: ${\gamma }_{01}^{-1}=84$ *μ*s, ${\gamma }_{12}^{-1}$ = 41 *μ*s, ${\gamma }_{23}^{-1}$ = 30 *μ*s, ${\gamma }_{34}^{-1}$ = 22 *μ*s, ${\gamma }_{\phi 1}^{-1}=72$ *μ*s, ${\gamma }_{\phi 2}^{-1}$ = 32 *μ*s, ${\gamma }_{\phi 3}^{-1}$ = 12 *μ*s, ${\gamma }_{\phi 4}^{-1}$ = 2 *μ*s, and *κ* ^−1^ = 15 *μ*s. The decoherence times of transmon qudits considered here are realistic because they are from the recent experimental report in ref. 11. In a realistic situation, it may be a challenge to obtain exact identical qudit-resonator couplings. Therefore, we consider inhomogeneous qudit-resonator couplings, e.g., *g*_1_ = *g* and *g*_2_ = 0.95 *g*.

**Figure 3:**
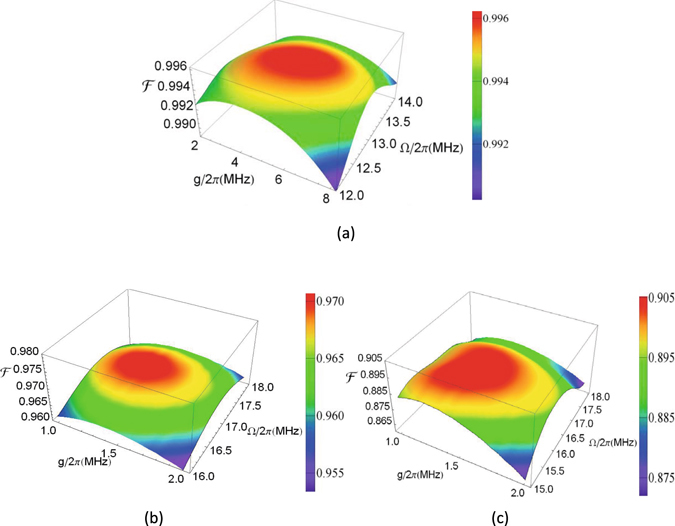
Fidelity for the quantum state transfer for *g*/2*π* and Ω/2*π*. (**a**) Fidelity for *d* = 3. (**b**) Fidelity for *d* = 4. (**c**) Fidelity for *d* = 5.

We numerically calculate the fidelity of the entire operation based on the master equation. Figure 3(a,b,c) shows the fidelity versus *g*/2*π* and Ω/2*π* for QST between two qudits for *d* = 3, *d* = 4, and *d* = 5, respectively. From Fig. 3(a), one can see that for *g*/2*π* ∈ [2, 8] MHz and Ω/2*π* ∈ [12, 14] MHz, the fidelity can be greater than 98.8% for *d* = 3. When *g*/2*π* = 5.4 MHz and Ω/2*π* = 12.8 MHz, the fidelity value is the optimum with a value of ~99.6% for *d* = 3. As shown in Fig. 3(b), the value of the fidelity has a slow decline for *d* = 4. In Fig. 3(b) the optimal value for ${\mathcal F}$~96.96% is obtained for *g*/2*π* =  = 1.35 MHz and for Ω/2*π* = 17.00 MHz. While ${\mathcal F}$ drastically decreases for *d* = 5, a high fidelity ~90.32% is attainable with *g*/2*π* = 1.45 MHz and Ω/2*π* = 16.00 MHz [see Fig. 3(c)]. Note that the above values of the *g* and Ω are readily available in experiments^64–67^.

**Figure 4:**
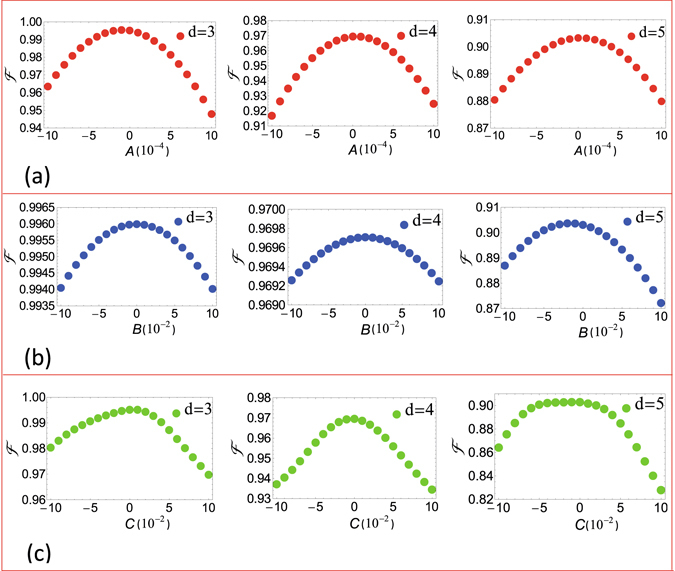
Fidelity versus *A*, *B*, and *C* for *d* = 3, *d* = 4, and *d* = 5, respectively. (**a**) Fidelity versus *A*. (**b**) Fidelity versus *B*. (**c**) Fidelity versus *C*.

To investigate the effect of the pulse errors on the fidelity of the QST, we consider a small frequency error *Aω*, a small phase error *Bϕ*, and a small duration error *Ct* of each pulse. The frequency, initial phase, and duration {*ω*, *ϕ*, *t*} of the pulses are thus modified as {*ω* + *Aω*, *ϕ* + *Bϕ*, *t* + *Ct*}, where the *ω*, *ϕ*, and *t* can be found for each of the pulses applied during the QST, as described in Section Results. With this modification, we numerically calculate the fidelity and plot Fig. 4, which shows how the fidelity of the QST varies with parameters *A*, *B*, and *C*. The values of *g* and Ω used in Fig. 4 are the ones just mentioned above, corresponding to the optimum fidelities in Fig. 3 for *d* = 3, *d* = 4, and *d* = 5, respectively. Other parameters used in the numerical simulation for Fig. 4 are the same as those used in Fig. 3. Figure 4(a) shows that the effect of the pulse frequency error on the fidelity is negligibly small for *A* ∈ [−10^−4^, 10^−4^], which corresponds to the pulse frequency error *Aω* ∈ [−10^−4^ *ω*, 10^−4^ *ω*]. Figure 4(b) shows that for *d* = 3 and *d* = 4, the fidelity is almost unaffected by the pulse phase error for *B* ∈ [−2 × 10^−2^, 2 × 10^−2^]; and for *d* = 5 the fidelity has a small decrease for *B* ∈ [−5 × 10^−2^, 2 × 10^−2^]. Figure 4(c) shows that the effect of the pulse duration error on the fidelity is negligible for *C* ∈ [−2 × 10^−2^, 2 × 10^−2^] for *d* = 3, *d* = 4 and *C* ∈ [−5 × 10^−2^, 2 × 10^−2^] for *d* = 5. These results indicate that compared to the phase error and the duration error, the fidelity is more sensitive to the pulse frequency error. From Fig. 4, one can see that the QST with high fidelity can be achieved for small errors in pulse frequency, phase, and duration.

For a cavity with frequency *ω*_*c*_/2*π* = 4.97 GHz and dissipation time *κ* ^−1^ used in the numerical simulation, the quality factor of the cavity is $Q\sim 4.7\times {10}^{5}$. Note that three-dimensional cavities with a loaded quality factor *Q* > 10^6^ have been implemented in experiments^64, 68^.

## Discussion

We have presented a method to deterministically transfer arbitrary *d*-dimensional quantum states (known or unknown) between two superconducting transmon qudits, which are coupled to a single cavity or resonator. As shown above, only a single cavity or resonator is needed, thus the experimental setup is very simple and the inter-cavity crosstalk is avoided. The state transfer can be performed by employing resonant interactions only. In addition, no measurement is required. Numerical simulation shows that high-fidelity transfer of quantum states between two transmon qudits for (*d* ≤ 5) is feasible with current circuit-QED technology. This proposal can be extended to transfer an arbitrary *d*-dimension quantum state between “ladder-type level structure” natural atoms (e.g., Rydberg atoms) or other artificial atoms (e.g., superconducting Xmon qudits, phase qudits, quantum dots), by employing a single cavity only.

The number of pulses can be reduced at a cost of using more than one cavity coupled to the qudits. However, the QST experimental setup will become complicated and the inter-cavity crosstalk is an issue, if two or more cavities are employed instead of a single cavity. Realistic QIP may not involve a large *d*. To the best of our knowledge, none of experimental works on QIP with qudits of *d* > 3 has been reported. In this sense, we think that this work is of interest. We hope this work will stimulate experimental activities in the near future.

## Methods

### Hamiltonian and time evolution

Consider two qudits 1 and 2 coupled by a cavity. The cavity is resonant with the transition between the two levels |0〉 and |1〉 of each qudit. In the interaction picture, the Hamiltonian is given by (in units of *ħ* = 1)

14 $${H}_{I\mathrm{,1}}=\sum _{j=1}^{2}\,{g}_{j}(a{\sigma }_{{\mathrm{01,}}_{j}}^{+}+h\mathrm{.}c\mathrm{.}),$$

where *a* is the photon annihilation operator for the cavity, the subscript *j* represents qudit *j*, ${\sigma }_{{\mathrm{01,}}_{j}}^{+}=\mathrm{|1}{\rangle }_{j}\langle \mathrm{0|}$, and *g*_*j*_ is the coupling constant between the cavity and the |0〉 ↔ |1〉 transition of qudit *j* (*j* = 1, 2). For simplicity, we set *g*_1_ = *g*_2_ ≡ *g*, which can be achieved by a prior design of qudits or adjusting the position of each qudit located at the cavity.

Under the Hamiltonian (14), one can obtain the following state evolutions:

15 $$\begin{array}{rcl}\mathrm{|0}{\rangle }_{1}\mathrm{|0}{\rangle }_{c}\mathrm{|0}{\rangle }_{2} & \to  & \mathrm{|0}{\rangle }_{1}\mathrm{|0}{\rangle }_{c}\mathrm{|0}{\rangle }_{2},\\ \mathrm{|1}{\rangle }_{1}\mathrm{|0}{\rangle }_{c}\mathrm{|0}{\rangle }_{2} & \to  & \frac{1}{2}(1+\,\cos \,\sqrt{2}gt)\mathrm{|1}{\rangle }_{1}\mathrm{|0}{\rangle }_{c}\mathrm{|0}{\rangle }_{2}\\  &  & -\,\frac{\sqrt{2}}{2}i\,\sin (\sqrt{2}gt\mathrm{)|0}{\rangle }_{1}\mathrm{|1}{\rangle }_{c}\mathrm{|0}{\rangle }_{2}\\  &  & -\,\frac{1}{2}\mathrm{(1}-\,\cos \,\sqrt{2}gt\mathrm{)|0}{\rangle }_{1}\mathrm{|0}{\rangle }_{c}\mathrm{|1}{\rangle }_{2},\end{array}$$

where |0〉_*c*_ (|1〉_*c*_) represents the vacuum (single photon) state of the cavity and subscript 1 (2) represents qudit 1 (2).

We now consider applying a classical pulse to a qudit, which is resonant with the transition between the level |*l* − 1〉 and the higher-energy level |*l*〉 of the qudit (*l* = 1, 2, …, *d* − 1). The Hamiltonian in the interaction picture is expressed as

16 $${H}_{I,l}={\rm{\Omega }}({e}^{i\phi }|l-1\rangle \langle l|+h\mathrm{.}c\mathrm{.}),$$

where Ω and *ϕ* are the Rabi frequency and the initial phase of the pulse. One can obtain the following rotations under the Hamiltonian (16),

17 $$\begin{array}{c}|l-1\rangle \to \,\cos ({\rm{\Omega }}t)|l-1\rangle -i{e}^{-i\varphi }\,\sin ({\rm{\Omega }}t)|l\rangle ,\\ |l\rangle \,\to cos({\rm{\Omega }}t)|l\rangle -i{e}^{i\phi }sin({\rm{\Omega }}t)|l-1\rangle \mathrm{.}\end{array}$$

The results given in Eqs (15) and (17) will be employed for implementing QST between two qudits, which is described in the Section Results.
